# Relationship between oral microbiota and colorectal cancer: A systematic review

**DOI:** 10.1111/jre.13289

**Published:** 2024-05-22

**Authors:** Sara Camañes‐Gonzalvo, José María Montiel‐Company, Miriam Lobo‐de‐Mena, María José Safont‐Aguilera, Amaya Fernández‐Diaz, Andrés López‐Roldán, Vanessa Paredes‐Gallardo, Carlos Bellot‐Arcís

**Affiliations:** ^1^ Department of Stomatology, Faculty of Medicine and Dentistry University of Valencia Valencia Spain; ^2^ Medical Oncology Department Consortium of the General University Hospital of Valencia, University of Valencia Valencia Spain; ^3^ Medical Oncology Department Requena General Hospital Valencia Spain

**Keywords:** colorectal cancer, oral microbiota, oral microorganisms, oral pathogens, periodontal pathogens

## Abstract

This systematic review aims to investigate the microbial basis underlying the association between oral microbiota and colorectal cancer. A comprehensive search was conducted across four databases, encompassing potentially relevant studies published up to April 2024 related to the PECO question: “Is there a differentiation in oral microbial composition between adult patients diagnosed with colorectal cancer compared to healthy patients?”. The Newcastle‐Ottawa Scale was used to evaluate the quality of the studies included. The level of evidence was assessed through the GRADE (Grading of Recommendations, Assessment, Development and Evaluation) tool. Sixteen studies fulfilled the eligibility criteria. Based on low to moderate evidence profile, high levels of certain subspecies within *Firmicutes* (such as *Streptococcus anginosus*, *Peptostreptococcus stomatis*, *S. koreensis*, and *S. gallolyticus*), *Prevotella intermedia*, *Fusobacterium nucleatum*, and *Neisseria oralis* were found to be associated with colorectal cancer. Conversely, certain bacteria (e.g., *Lachnospiraceae*, *F. periodonticum*, and *P. melaninogenica*) could exert a symbiotic protective effect against colorectal cancer. Based on existing evidence, it appears that variations in oral microbiota composition exist among individuals with and without colorectal cancer. However, further research is necessary to determine the mechanisms of oral dysbiosis in colorectal carcinogenesis.

## Graphical abstract

High levels of certain bacteria such as *Lachnospiraceae*, *F. periodonticum*, and *P. melaninogenica* could exert a symbiotic protective effect against colorectal cancer. On the contrary, an increase in subspecies within Firmicutes (such as *Streptococcus anginosus*, *Peptostreptococcus stomatis*, *S. koreensis*, and *S. gallolyticus*), *Prevotella intermedia*, *Fusobacterium nucleatum*, and *Neisseria oralis* were found to be associated with colorectal cancer.

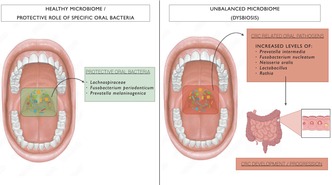

## 1 INTRODUCTION

Cancers within the gastrointestinal tract (GI), include esophageal cancer (EC), gastric cancer (GC), colon cancer, rectal cancer, liver cancer (LC), pancreatic cancer (PC), biliary tract cancer, and small bowel cancer. In 2020, these tumors accounted for 26.3% of cancer cases, contributing to 35.4% of cancer‐related deaths worldwide.^1^ Notably, colorectal cancer (CRC) emerged as the most prevalent GI cancer, representing 38.8% of GI cancer cases and causing 26.0% of GI cancer‐related deaths, followed by GC (21.7% of cases and 23.2% of deaths), LC (17.9%/23.2%), EC (12.2%/15.0%), and PC (9.5%/12.7%).^1^, ^2^

The microbiota, encompassing the genes within its microbiome, plays a vital role in maintaining human health.^3^, ^4^ Advances in metagenomics have unveiled that microhabitats exist, distributed throughout the human body, each hosting a unique ecosystem characterized by distinct atmospheric and nutritional conditions that nurture symbiotic relationships.^5^ However, this dynamic equilibrium can be disrupted by environmental factors and external interferences, such as antibiotic usage,^4^ often resulting in dysbiosis, an imbalance in bacterial communities. Dysbiosis has been linked to a spectrum of pathological conditions, including cancer.^5^, ^6^

The human oral microbiome has been relatively understudied in comparison to the gut microbiome. It comprises over 770 species‐level taxa, each prevalent in different ecosystems, with various subtypes.^7^ Culture‐independent molecular techniques, like 16S rRNA gene sequencing, have been used extensively to classify the oral microbiome.^8^, ^9^ An invaluable resource in this field is the Human Oral Microbiome Database (HOMD), which provides definitions for oral bacterial taxa and database of oral bacterial genome sequences.^10^ The HOMD encompasses 16S rRNA genes from 15 phyla within the oral bacterial domain, with the six most prominent phyla being *Firmicutes*, *Actinobacteria*, *Proteobacteria*, *Bacteroidetes*, *Fusobacteria*, and *Spirochaetes*.^8^, ^11^

Emerging research suggests a potential association between the oral microbiota and the development of CRC, both through direct and indirect mechanisms.^12^, ^13^, ^14^ Periodontitis has been linked to an increased risk of various chronic non‐communicable diseases, including CRC. Indeed, dysbiosis of the oral microbiota and immune‐inflammatory pathways related to periodontitis may impact the pathophysiology of the gastrointestinal tract and its accessory organs through the so‐called “gum–gut axis.” In addition to the hematogenous spread of periodontal pathogens and inflammatory cytokines, recent research suggests that oral pathobionts may translocate to the gastrointestinal tract through saliva, possibly influencing neoplastic processes in the gastrointestinal systems. The exact mechanisms by which oral pathogens contribute to the development of digestive tract cancers are not fully understood but may involve dysbiosis of the gut microbiota, chronic inflammation, and immune modulation/evasion, mainly through the interaction with T‐helper and monocytic cells. Specifically, keystone periodontal pathogens, including *Porphyromonas gingivalis* and *Fusobacterium nucleatum*, are known to interact with the molecular hallmarks of gastrointestinal cancers, inducing genomic mutations, and promoting a permissive immune microenvironment by impairing anti‐tumor checkpoints.^15^, ^16^ Furthermore, individuals exhibiting inadequate oral hygiene were found to be at a higher likelihood of harboring these periodontal pathogens within the oral cavity.^17^

To elucidate the biological plausibility underlying the potential association between oral microbiota and CRC, it is imperative to delve into mechanistic insights supported by existing literature. Specific oral bacteria, such as *Porphyromonas*, *Prevotella*, and *Fusobacterium*, have been identified in association with carcinogenic processes. These bacteria can induce a chronic inflammatory response by stimulating the production of pro‐inflammatory cytokines like interleukin‐1β, interleukin‐6, and matrix metalloproteinases.^18^ Moreover, *Prevotella* and *Fusobacterium* have been implicated in carcinogenesis through mechanisms involving the suppression of host immune functions and facilitation of malignant transformation in epithelial cells.^18^

In this context, recent evidence suggests that specific oral bacteria could serve as biomarkers for the early detection of colorectal cancer, potentially offering a non‐invasive screening approach.^19^, ^20^, ^21^, ^22^ However, it is important to evaluate and differentiate the periodontal status presented by individuals, as the presence of oral diseases, particularly periodontitis, impacts the composition of oral microbiota.^23^, ^24^, ^25^

Considering the potential of the oral microbiota as a non‐invasive alternative for population‐wide screening and the diagnosis of CRC, this systematic review was conducted to provide an up‐to‐date assessment of the existing literature on the efficacy of these oral bacteria in distinguishing individuals with CRC from those without the condition. Additionally, the periodontal status of the patients included in the analyzed articles has been taken into account.

## 2 METHODS

This systematic review adhered to the 2020 PRISMA (Preferred Reporting Items for Systematic Reviews and Meta‐Analysis) guideline update.^26^

### 2.1 PECO question

The aim was to ascertain whether an association exists between the presence of specific oral bacteria and the diagnosis of CRC, guided by the PECO (Population/Patient, Exposure, Comparison, Outcome) question: Is there a distinction in oral microbial composition between adult patients diagnosed with CRC and healthy patients? Thus, the exposure in this research was CRC, the outcome was the composition of the oral microbiota, and the comparison involved patients without CRC.

### 2.2 Inclusion and exclusion criteria

The inclusion criteria for the study encompassed “Articles” and “Articles in press,” including longitudinal studies, retrospective and prospective case–control studies, without restrictions on the year of publication or language. The inclusion criteria comprised: (1) a study population diagnosed with colorectal cancer; (2) studies encompassing both male and female genders; (3) studies involving patients from whom an oral sample is taken containing oral bacterial species and pathogens, examining the potential association between oral microbiota and CRC; (4) studies including a healthy control group; (5) studies that analyze the oral microbiota regardless of the microbiological technique employed; (6) studies that assess oral and periodontal health status. Studies that did not address the PECO question were excluded.

### 2.3 Search strategy

A comprehensive electronic search was conducted across the Medline (PubMed), Excerpta Medica database (EMBASE), Scopus, and Web of Science databases to identify potentially pertinent studies, regardless of language barriers. Additionally, the references of the included studies underwent manual scrutiny to identify articles meeting the inclusion criteria but not found in the aforementioned databases. The search was last updated in April 2024.

### 2.4 Search terms

The search strategy incorporated key terms such as “oral microbiota”, “oral bacteria”, “oral microorganisms”, “periodontal pathogens”, “oral pathogens”, “periodontal disease”, “periodontitis”, “oral microbiome”, “digestive tract cancer”, “biomarker”, “gastrointestinal cancer”, “digestive cancer”, “colonic cancer”, “digestive system cancer,” “colorectal cancer,” “patient,” and “cancer patient.” Boolean operators (“OR” and “AND”) were employed to connect terms relevant to the research question.

These keywords were categorized into three groups, and a comprehensive exploration of all conceivable combinations among the terms in these three groups was conducted. The identified articles were then imported into Mendeley Desktop 1.13.3 software (Mendeley Ltd, London, England) to identify and eliminate duplicates. The complete search strategy for all databases is provided in the Table S1.

### 2.5 Selection process

Two independent reviewers (SCG and MLDM) systematically evaluated the titles and abstracts of all identified articles. In cases of disagreement, a third reviewer (MJSA) was consulted. If the abstract lacked sufficient information for a decision, the reviewers examined the full‐text article before arriving at a final determination. During the second phase of study selection, the same reviewers scrutinized the full‐text articles, documenting reasons for the exclusion of articles based on the inclusion and exclusion criteria.

### 2.6 Study data

The exposure is defined as the potential association between oral microbiota and CRC, focusing on patients diagnosed with this pathology. Information regarding the type and characteristics of the patient populations across all studies was meticulously gathered, including gender, age, country of study, details regarding antibiotic use or prior CRC treatment before sample collection, as well as an assessment of periodontal health status, CRC stage and information about potential treatments received previously for CRC. The evaluation of oral microbiota composition involves comprehensive details on sample collection specifics, such as bio‐specimen source, collection time, storage temperature, method of detection, genome extraction, database utilization, and data processing protocols. Accepted microbiological analyses encompass a wide range of methodologies, ranging from culture‐dependent techniques to genome sequencing approaches.

### 2.7 Quality assessment

The quality of the studies was independently assessed by the same investigators using the Newcastle‐Ottawa Scale, a tool for evaluating the methodological quality of meta‐analyses of observational studies.^27^ Any discrepancies between the two investigators were resolved through consensus, with a third investigator consulted in case of uncertainty. The GRADE tool for formulating and grading recommendations in clinical practice was also employed.^28^

## 3 RESULTS

### 3.1 Study selection and flowchart

**FIGURE 1:**
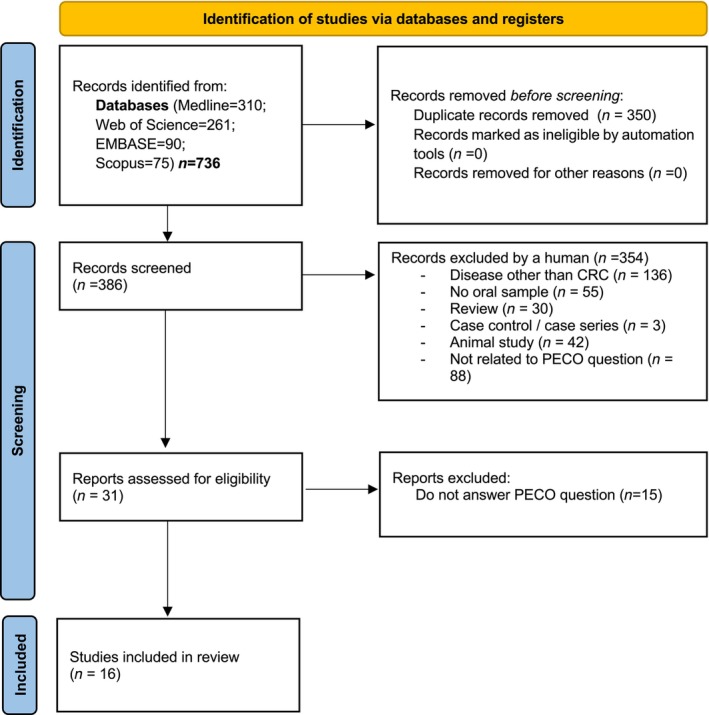
PRISMA flow diagram for systematic review which includes searches of databases.

The initial search yielded 736 references, with 310 in PubMed, 90 in EMBASE, 261 in Web of Science, and 75 in Scopus. After eliminating 350 duplicated articles, the remaining 386 were scrutinized. Of these, 354 were excluded based on the title and abstract, as they were unrelated to the research question. After a thorough examination of the full text of the resulting 31 articles, 15 were further excluded. Ultimately, 16 articles met the eligibility criteria and underwent qualitative review. The PRISMA flowchart in Figure 1 provides a comprehensive overview of the article selection process.

### 3.2 Quality assessment

The selected studies comprised 16 case–control studies.^12^, ^22^, ^29^, ^30^, ^31^, ^32^, ^33^, ^34^, ^35^, ^36^, ^37^, ^38^, ^39^, ^40^, ^41^, ^42^ Most articles demonstrated high quality on the Newcastle‐Ottawa scale (Table S2; Figure S1). The lowest evidence score was 6 out of 9, yet the majority of the included studies scored above 8 out of 9, indicating a high level of evidence. The GRADE tool for formulating and grading recommendations demonstrates an evidence profile ranging from very low to moderate (Table S3).

### 3.3 Characteristics of population in the included studies

An overview of the included studies is provided in Table 1. The selected studies, conducted between 2016 and 2023, comprised 16 studies conducted in various regions. Nine of the articles were conducted in Asia,^30^, ^32^, ^33^, ^34^, ^37^, ^38^, ^39^, ^42^ two in the USA,^29^, ^36^ two in Canada,^40^, ^41^ and four in Europe.^12^, ^31^, ^33^, ^35^

**TABLE 1:** Association between colorectal cancer (CRC) and oral microbiome.

Study	Bio‐specimen source	Collection time	Country	Temperature for storage	Database	Method of detection
Kato et al. (2016)^29^	Oral rinse	2003–2005	USA	−80°C	HOMD	16S rRNA V3‐V4
Han et al. (2016)^30^	Tongue coating	2013–2015	China	N.A.	Mothur and SILVA	16S rRNA V4
Russo et al. (2017)^31^	Unstimulated saliva	2015–2016	Italy	−80°C	SINA standalone classifier; “Ref NR 99”	16S rRNA; qPCR
Yamamura et al. (2017)^32^	Swab (cheeks)	N.A.	Japan	N.A.	N.A.	qPCR
Flemer et al. (2018)^12^	Swab (cheeks)	N.A.	Ireland	−80°C	Mothur and RDP	16S rRNA V3‐V4; qPCR
Guven et al. (2019)^33^	Unstimulated saliva (5 mL)	N.A.	Turkey	−20°C	N.A.	qPCR
Kageyama et al. (2019)^34^	Stimulated saliva	2015–2017	Japan	−80°C	HOMD	16S rRNA V1‐V2
Schmidt et al. (2019)^35^	Saliva	N.A.	France	−80°C	PATRIC	16S rRNA sequencing
Yang et al. (2019)^36^	Oral Rinse	2002–2009	USA	N.A.	HOMD	16S rRNA V4
Zhang et al. (2020)^37^	Swab (cheeks)	N.A.	China	−80°C	Mothur and RDP	16S rRNA V3‐V4
Uchino et al. (2021)^38^	Saliva	N.A.	Japan	−4°C	NCBI	16S rRNA sequencing
Wang et al. (2021)^39^	Unstimulated saliva	2017–2018	China	−80°C	Mothur and SILVA	16S rRNA sequencing
Idrissi Janati et al. (2022)^40^	Saliva	2018–2019	Canada	−80°C	N.A.	qPCR
Morsi et al. (2022)^41^	Saliva	N.A.	Canada	−80°C	Uniprot protein	Spectrometry
Zhang et al. (2022)^42^	Swab (cheeks)	N.A.	China	−80°C	SILVA	16S rRNA V4
Rezasoltani et al. (2023)^22^	Saliva	2020–2021	Iran	−80°C	SILVA	16S rRNA; qPCR

*Note*: Characteristics of sample collection and measurement in the included studies.

Abbreviations: HOMD, human oral microbiome database; IMM, indirect immunofluorescence microscopy; N.A., not applicable; NCBI, National Center for Biotechnology Information; qPCR, quantitative polymerase chain reaction; RDP, ribosomal database project.

Table 2 summarizes the general characteristics of the population examined. All studies delineated differences in oral microbiota composition between cases (CRC patients) and controls. The total sample analyzed from the included studies consists of 954 cases and 1280 controls, with subjects ranging in age from 35 to 80 years old.

**TABLE 2:** Association between colorectal cancer (CRC) and oral microbiome.

Study	Sample size (Ca/Co)	Age (years)	Gender (M/F)	CRC stage	Antibiotic use prior to sample collection	Oncologic treatment prior to sample collection	Oral hygiene	Periodontal health
Kato et al. (2016)^29^	68/122	40–80	Ca 32/36 Co 41/81	N.A.	N.A.	No oncologic treatment	N.A.	N.A.
Han et al. (2016)^30^	90/100	55.45 ± 11	Ca 43/47 Co 51/49	I: 16; II: 38; III: 28; IV: 8 (AJCC)	N.A.	N.A.	N.A.	N.A.
Russo et al. (2017)^31^	10/10	71–95	Ca 4/6 Co 6/4	TMN I:3; II:2; IV:4; IV 1	Not in 12 weeks	No oncologic treatment	Addressed (OHI)	Presence of periodontitis (PPD)
Yamamura et al. (2017)^32^	20/20	40–80	N.A.	Cancer manual staging	N.A.	No oncologic treatment	N.A.	N.A.
Flemer et al. (2018)^12^	45/25	58.6 ± 12.4	Ca 56.1 M Co 37.5 M	N.A.	Not in 4 weeks	N.A.	N.A.	N.A.
Guven et al. (2019)^33^	71/77	56 ± 10	Ca 46/25 Co 37/40	I:4; II 16; III: 24; IV: 27 (AJCC)	Not in 4 weeks	No oncologic treatment	N.A.	N.A.
Kageyama et al. (2019)^34^	24/118	38–84	Ca 16/8 Co 84/34	N.A.	Not in 4 weeks	No oncologic treatment	Addressed	Presence of periodontitis (PPD; BOP)
Schmidt et al. (2019)^35^	53/88	40–80	Ca 25/28 Co 40/48	N.A.	N.A.	N.A.	N.A.	N.A.
Yang et al. (2019)^36^	231/462	40–79	Ca 93/138 Co 185/276	Incident CRC	Not in 1 week	No oncologic treatment	Addressed (OHI)	Presence of periodontitis/gingivitis (BOP)
Zhang et al. (2020)^37^	161/58	59.25 ± 10	Ca 107/54 Co 31/27	I: 24; II: 66; III 60; IV: 11 (AJCC)	N.A.	No oncologic treatment	N.A.	N.A.
Uchino et al. (2021)^38^	52/51	68.52 ± 10	Ca 33/19 Co 26/25	I, II: 26; III, IV: 26 (AJCC)	Not in 1 week	No oncologic treatment	Addressed (OHI)	Presence of periodontitis/gingivitis (BOP)
Wang et al. (2021)^39^	30/30	35–80	Ca 17/13 Co 15/15	N.A.	Not in 1 week	No oncologic treatment	Addressed (OHI)	Presence of periodontitis/gingivitis
Idrissi Janati et al. (2022)^40^	22/21	40–80	Ca 18/4 Co 10/11	Stage I and Stage II (AJCC)	Not in 12 weeks	No oncologic treatment	Addressed	Presence of periodontitis/gingivitis
Morsi et al. (2022)^41^	4/8	40–80	Ca 3/1 Co 4/4	N.A.	Not in 12 weeks	No oncologic treatment	Addressed	N.A.
Zhang et al. (2022)^42^	53/70	59.21 ± 14	Ca 37/16 Co 36/34	I, II: 23 III, IV: 30 (AJCC)	Not in 4 weeks	No oncologic treatment	Addressed	N.A.
Rezasoltani et al. (2023)^22^	20/20	40–80	N.A.	Stage 0 and Stage I (AJCC)	Not in 12 weeks	No oncologic treatment	N.A.	N.A.

*Note*: Characteristics of population in the included case–control studies.

Abbreviations: AJCC, American Joint Committee on Cancer; BOP, bleeding on probing; CRC, colorectal cancer; N.A., not applicable; not addressed; PPD, periodontal pocket depth; TMN, tumor node metastasis classification.

Understanding the CRC stage of the study population is crucial. Ten of the 16 included studies described or classified patients based on disease stage (Table 2). For patient disease stage classification, the authors utilized the *American Joint Committee on Cancer* classification.

The composition and diversity of oral microbiota can be significantly influenced by the administration of antibiotics. Eleven studies excluded participants who had used antibiotics within 1–12 weeks preceding the sample collection period.^12^, ^22^, ^31^, ^33^, ^34^, ^36^, ^37^, ^38^, ^39^, ^40^, ^41^ Conversely, the remaining studies did not provide information regarding antibiotic usage by the participants. It is noteworthy that variations in the quantity, complexity, and quality of oral microbiota also occur during cancer treatment. Thirteen of the included studies explicitly mentioned the exclusion of participants undergoing oncologic treatment prior to sample collection.^12^, ^22^, ^31^, ^32^, ^33^, ^34^, ^36^, ^37^, ^38^, ^39^, ^40^, ^41^, ^42^

Regarding the periodontal health status, six out of the 16 included studies conducted an assessment of periodontal health.^31^, ^34^, ^36^, ^38^, ^39^, ^40^ To address this, the studies determined whether individuals had periodontal disease or not, and of what type (gingivitis or periodontitis), through clinical variables. Furthermore, eight studies assessed whether patients had good oral hygiene habits employing a series of indices^31^, ^34^, ^36^, ^38^, ^39^, ^40^, ^41^, ^42^ (Table 2).

### 3.4 Characteristics of sample collection and measurement

An overview of the sample information is provided in Table 1. All samples were sourced from the oral cavity, predominantly from unstimulated and stimulated saliva, obtained through oral rinse and swabbing (inside both cheeks). The majority of frozen samples were stored at temperatures below −80°C or −70°C, with only two studies opting for storage at temperatures of −20°C or lower before analysis.^33^, ^38^

In the analysis of oral bacterial phylogeny and taxonomy, 12 studies employed 16S rRNA gene sequencing.^12^, ^22^, ^29^, ^30^, ^31^, ^34^, ^35^, ^36^, ^37^, ^38^, ^39^, ^42^ Six studies utilized quantitative polymerase chain reaction (qPCR) to quantify the abundance of specific oral bacteria,^12^, ^22^, ^31^, ^32^, ^33^, ^40^ and one study employed spectrometry.^41^

### 3.5 Quantitative synthesis

Table 3 delineates the principal differences in increased bacteria between the case and control groups.

**TABLE 3:** Association between colorectal cancer (CRC) and oral microbiome.

Article	Bacteria	
	Increased in cases	Increased in controls
Kato et al. (2016)^29^	**Firmicutes** (*Lactobacillus*) **Actinobacteria** (*Rothia*)	
Han et al. (2016)^30^	**Bacteroidetes** (*Prevotella salivae*)	**Fusobacteria** (*Fusobacterium periodonticum*) **Bacteroidetes** (*Prevotella aurantiaca*)
Russo et al. (2017)^31^	N.S.	
Yamamura et al. (2017)^32^	**Fusobacteria** (*F. Nucleatum*)	
Flemer et al. (2018)^12^	**Actinobacteria** (*Actinomyces*, *Rothia*) **Firmicutes** (*Veillonella*, *Streptococcus*, *Parvimonas micra* – Peptostreptococcus – *Peptostreptococcus stomatis, Dialister pneumosintes* – veillonella‐ *Lachnoanaerobaculum* ‐eubacterium‐) **Proteobacteria** (*Haemophilius, Neisseria*) **Bacteroidetes** (*Prevotella*, *Alloprevotella*) **Fusobacteria** (*F. Nucleatum*)	*Lachnospiraceae* (Anaerostipes, Blautia, Roseburia)
Guven et al. (2019)^33^	**Fusobacteria** (*F. Nucleatum*) **Firmicutes** (*Streptococcus gallolyticus/bovis*)	
Kageyama et al. (2019)^34^	**Bacteroidetes** (*Porphyromonas gingivalis*) **Actinobacteria** (*Actinomyces odontolyticus, Corynebacterium*) **Proteobacteria** (*Neisseria*)	**Bacteroidetes** (*Prevotella melaninogenica*, *Porphyromonas pasteri*) **Firmicutes** (*Streptococcus* sp.)
Schmidt et al. (2019)^35^	**Firmucutes** (*Streptococcus anginosus, Veilonella atípica*, *Peptostreptococcus stomatis, Solobacterium moorei* ‐eubacterium‐).	
Yang et al. (2019)^36^	**Bacteroidetes** (*Prevotella intermedia, prevotella denticola, prevolella* sp. *oral taxon 300*) **Spirochaetes** (*Treponema denticola*) **Actinobacteria** (*Bifidobacterium dentium*) **Firmicutes** (*Lactobacillus salivarius, Eubacterium yurii, Peptococcus*) **Proteobacteria** (*Neisseria, Campylobacter* sp.)	**Bacteroidetes** (*Prevotella melaninogenica*). **Firmicutes**: (*Carnobacteriaceae, Streptococcaceae, Erysipelotrichaceae, Streptococcus* sp. *oral taxon 058, Streptococcus* sp. *Solobacterium moorei*)
Zhang et al. (2020)^37^	**Fusobacteria** (*Fusobacterium*) **Bacteroidetes** (*Prevotella*) **Firmicutes** (*Veillonellla*) **Spirochaetes** (*Treponema*) **Bacteroidetes** (*Phorphyromonas*)	
Uchino et al. (2021)^38^	**Firmicutes** (*Peptostreptococcus stomatis, Streptococcus anginosus, Solobacterium moorei, Streptococcus koreensis*) **Fusobacteria** (*nucleatum* subsp. *nucleatum*)	
Wang et al. (2021)^39^	**Firmicutes** (*Streptococcus*) **Bacteroidetes** (*Porphyromonas gingivalis, Prevotella*) **Proteobacteria** (*Haemophilius, Neisseria*)	**Fusobacteria** (*Fusobacterium periodonticum*)
Idrissi Janati et al. (2022)^40^	**Fusobacteria** (*F. nucleatum*)	
Morsi et al. (2022)^41^	N.S (*F. nucleatum* subsp.)	
Zhang et al. (2022)^42^	**Firmicutes** (*Streptococcus*¸ *Herbaspirillum*)	**Proteobacteria** (*Haemophilus Neisseria*) **Fusobacteriota**
Rezasoltani et al. (2023)^22^	**Firmicutes** (*Eubacterium* spp.) **Actinobacteria** (*Bifidobacterium* spp.) **Fusobacteria** (*Fusobacterium* spp.)	

Abbreviation: N.S., non‐significant.

#### 3.5.1 *Firmicutes* phylum

Concerning *Firmicutes* phylum levels, ten studies reported an elevated level in cases compared to controls.^12^, ^22^, ^29^, ^33^, ^35^, ^36^, ^37^, ^38^, ^39^, ^42^ Within the *Firmicutes* phylum, specifically the genus *Lactobacillus*, Kato et al. established an association between a history of CRC and an approximately two‐fold increase in the presence of this genus (95% CI 1.08–4.00).^29^ Additionally, Yang et al. identified a significant association, indicating a 1.46‐fold increase (95% CI 1.03–2.08; GRADE tool: very low profile).^36^

On the contrary, regarding the genus *Eubacteria*, also encompassed within the phylum *Firmicutes*, Schmidt et al. noted an elevated level of *Solobacterium moorei* in cases compared to control patients.^35^ However, Yang et al. reported opposing results regarding the association between this bacterium and CRC, with an odds ratio of 0.87 (95% CI 0.77–0.99).^36^ Conversely, a symbiotic interaction has been suggested for certain species within the *Firmicutes* phylum. Flemer et al. discovered a negative association between the colonic presence and abundance of oral pathogens and the colonic abundance of *Lachnospiraceae*, a component of *Eubacteria*.^12^

In relation to the genus *Streptococcus*, six studies have reported increased levels of specific species in CRC patients (GRADE tool: low‐moderate profile).^12^, ^33^, ^35^, ^38^, ^39^, ^42^ However, Russo et al. found no statistically significant differences.^31^ Notably, the study by Guven et al. demonstrated that the quantity of *Streptococcus gallolyticus* (Sg) in saliva was higher in the patient group compared to controls (4.12 ± 0.99 vs. 3.15 ± 0.58 log10 copies/mL, *p* < .001).^33^ Moreover, the abundance of this bacterium was found to have diagnostic value for CRC (AUC 0.84; 95% CI: 0.72–0.96; *p* < .001). A salivary quantity of *S. gallolyticus* above 3.32 log10 copies/mL emerged as the optimal predictor cut‐off value for CRC, with 82% sensitivity and 76% specificity.

#### 3.5.2 *Bacteroidetes* phylum

Concerning *Bacteroidetes* phylum levels, seven studies identified an elevated level in cases compared to controls.^12^, ^30^, ^33^, ^34^, ^36^, ^37^ Within the *Bacteroidetes* phylum, Yang et al. associated *Prevotella intermedia* with an increased risk of CRC, reporting an odds ratio (OR) of 1.55 (95% CI: 1.08–2.22).^36^ These findings align with results from other studies.^12^, ^30^, ^37^, ^39^ However, Yang et al. reported that *Prevotella melaninogenica* was associated with a decreased risk of CRC, with an OR (95% CI) of 0.91 (0.84–0.99),^36^ a trend consistent with the study by Kageyama et al.^34^ (GRADE tool: very low profile).

Within this phylum, Guven et al. found that the quantity of *Porphyromonas gingivalis* (Pg) was comparable in the case and control groups (4.28 ± 1.72 vs. 4.12 ± 1.24 log10 copies/mL, *p* = .837).^33^ These results were in concordance with findings from other studies.^34^, ^39^

#### 3.5.3 *Proteobacteria* phylum

Four studies have established a correlation between CRC and certain subspecies of the *Proteobacteria* phylum.^12^, ^34^, ^36^, ^39^ *Neisseria oralis* sp. was identified as associated with an increased risk of CRC, with an odds ratio (OR) of 1.42 (95% CI: 1.01–2.00), as reported by Yang et al.^36^ These findings align with results from other studies.^12^, ^34^, ^39^ Conversely, *Campylobacter* sp. *oral taxon 044* sp. was linked to an increased risk of CRC, with an OR (95% CI) of 1.58 (1.12–2.24), as reported by Yang et al.,^36^ following adjustments for smoking pack‐years, alcohol consumption status, and sequencing depth.

#### 3.5.4 *Fusobacteria* phylum

Five previous studies have consistently reported a potential association between *Fusobacterium nucleatum* (*F. nucleatum*), originating from the oral cavity, and CRC progression^12^, ^22^, ^33^, ^37^, ^40^ (GRADE tool: low‐moderate profile). Idrissi Janati et al. found that *F. nucleatum* levels, measured by qPCR as 2−ΔCq in saliva, ranged from barely detectable (0.000004) to 3.17 and 2.65 in cases and controls, respectively.^40^ The median (95% CI) salivary *F. nucleatum* level was 0.345 (0.15–0.82) in the case group and 0.12 (0.05–0.65) in the control group. A study in Turkey, with a substantial number of participants and employing qPCR for microbial saliva analysis, demonstrated a higher mean amount of *F. nucleatum* in the CRC group than in the control group (6.89 ± 1.07 in the case group vs. 6.35 ± 0.78 log10 copies/mL, *p* = .001).^33^

Conversely, *Fusobacterium periodonticum* levels were significantly different between CRC patients and healthy individuals, with higher levels observed in controls (1514.63 ± 1106.82 controls vs. 433.07 ± 544.04 CRC cases; *p* = .018; GRADE tool: very low profile).^30^

#### 3.5.5 *Actinobacteria* phylum

Within the *Actinobacteria* phylum, Kato et al. demonstrated an association between a history of CRC and an increased relative abundance of *Rothia* by 28% (1.28; 95% CI 1.02–1.59; GRADE tool: very low profile).^29^ Similar findings were reported by Flemer et al.^12^ However, this association was not observed in other studies.^36^ Additionally, other subspecies such as *Actinomyces* and *Bifidobacterium* have shown significantly higher levels in CRC patients compared to controls in various studies.^12^, ^22^, ^34^, ^36^ For instance, *Bifidobacterium* sp. was associated with an increased risk of CRC, with an odds ratio (95% CI) of 1.10 (1.01–1.19) and a *p* value of .03.^36^ These results are consistent with findings from other studies.^22^

#### 3.5.6 *Spirochaetes* phylum

Within the *Spirochaetes* phylum, the presence of *Treponema denticola* was associated with an increased risk of CRC, with an OR (95% CI) of 1.76 (1.19–2.60) and a *p* value of 4.45 × 10^−3^, as reported by Yang et al.^36^ These findings align with results from other studies.^37^

## 4 DISCUSSION

Colorectal cancer is a global health concern, with an estimated 147 950 new cases and 53 200 new deaths reported in the United States in 2020.^43^ In the oral cavity, a diverse bacterial community influences both oral and systemic diseases.^44^, ^45^, ^46^ As oral microbiota is swallowed, potentially reaching the digestive tract, it could be a risk factor for distant‐site cancers.

Non‐invasive CRC screening tools detect advanced carcinomas but lack sensitivity for early lesions.^47^ Conversely, oral microbiota proves highly reliable for CRC screening, with both high specificity and sensitivity.^12^, ^22^ Notably, the salivary microbiome exhibits greater stability than the gut microbiome.^48^ This review exclusively focuses on oral samples. It establishes a possible association between oral microbiota and CRC.

Within *Firmicutes*, CRC patients consistently exhibited elevated *Lactobacillus*,^29^, ^36^ *Solobacterium moorei*,^35^, ^38^ and *Streptococcus* ^12^, ^33^, ^35^, ^38^, ^39^, ^42^ levels compared to controls. The increased occurrence of these bacteria in the saliva of CRC patients, along with their notable predictive value for CRC,^33^ may have implications for screening, early diagnosis, and prevention if a potential association with CRC is confirmed. However, some evidence suggests a potential protective effect within this phylum. Flemer et al.^12^ found a negative association between the colonic presence and abundance of oral pathogens and the colonic abundance of *Lachnospiraceae*. Additionally, weak negative correlations of this bacteria with dietary habits reminiscent of a “Western diet” were detected. Hence, *Lachnospiraceae* may potentially protect against colonization with oral bacteria, potentially mediated through dietary habits (GRADE tool: very low profile).

Within *Bacteroidetes*, five studies indicate increased levels of *Prevotella intermedia* in CRC patients compared to controls.^12^, ^30^, ^36^, ^37^, ^39^ *Prevotella intermedia* can contribute to a chronic inflammatory reaction by stimulating the production of inflammatory mediators such as interleukin‐1β, interleukin‐6, and matrix metalloproteinases.^18^ This bacterium has also been linked to carcinogenesis through mechanisms involving the suppression of host immunological functions and the promotion of malignant transformation in epithelial cells.^18^ However, regarding *Porphyromonas gingivalis* (Pg), various studies show similar amounts in both cases and control groups.^33^, ^34^, ^39^ Increased Pg levels are observed in saliva samples of patients with pancreatic,^34^, ^49^ gastric,^34^ and esophageal cancer,^34^, ^50^ and the risk appears to increase with age.^51^ Indeed, Pg can negatively interact with colon carcinoma cells, possibly upregulating the inflammatory pathway pivoted by PD‐L1 as described also for oral squamous cell carcinoma and prostate cancer,^15^ but this association isn't clear in CRC patients.

In *Proteobacteria*, a correlation with CRC is reported with *Neisseria oralis* sp., which is capable of producing acetaldehyde linked to digestive tract cancer risk^12^, ^34^, ^36^, ^39^, ^52^ (GRADE tool: low profile). However, Zhang et al.^42^ found higher levels in controls, possibly due to in vitro observations not reflecting in vivo behavior amid bacterial community interactions.^53^ Elucidating acetaldehyde‐mediated carcinogenesis requires comprehensive in vitro and in vivo studies of *Neisseria* species.

Concerning *Fusobacteria*, seven studies indicated an association between *F. nucleatum* from the oral cavity and CRC progression.^12^, ^22^, ^32^, ^33^, ^37^, ^38^, ^40^ *F. nucleatum* has been identified among the most prevalent bacterial species in CRC tissues. Although the evidence for its translocation from the oral environment to the gut needs to be confirmed by strain‐level metagenomics techniques, the mechanistic role of *F. nucleatum* as the “alpha‐bug” with direct cancer‐accelerating features has been repeatedly suggested.^54^ Briefly, enhancement of cancer cell proliferation, establishment of a cancer‐promoting immune environment through miRNA‐mediated activation of TLR2/TLR4, and the evasion of immune checkpoints have been proposed as its main mechanisms.^15^ However, some studies fail to confirm the CRC‐*F. nucleatum* association.^29^, ^31^, ^34^, ^36^ This discrepancy may stem from *F. nucleatum*'s dependence on subspecies and strain variations. Uchino et al. found *F. nucleatum* subsp. *nucleatum* was significantly more abundant in CRC patients' saliva, while subsp. *vincentii* was higher in their stool.^38^ Despite both being *F. nucleatum*, they differ at the subspecies and strain levels, consistent with Komiya et al.'s findings.^55^ Hypothesized from these results, *F. nucleatum* subsp. *nucleatum* may colonize large intestine tissues, but transferring from the oral cavity to the intestine is challenging, with limited association on CRC progression. In contrast, *F. nucleatum* subsp. *vincentii* could consistently reach intestinal tissues, influencing CRC progression.

Within the *Actinobacteria* phylum, an increased relative abundance of *Rothia* has been linked to CRC.^12^, ^29^ Nevertheless, this association was not identified in other studies.^36^ Consequently, whether this bacterium promotes colorectal carcinogenesis remains debatable. The genus *Rothia* is typically considered a benign oral commensal. However, *Rothia* has recently been recognized as an opportunistic pathogen causing various diseases in immunocompromised hosts^56^ and has been associated with periodontitis in acquired immunodeficiency syndrome patients.^57^ Hence, an inflammatory microenvironment could potentially promote CRC development, but further comprehensive studies are required.

The results of the present systematic review, highlight the potential migration of periodontal pathogens toward the digestive tract, overcoming the stomach's acidic barrier, typically protective against intestinal infections. This migration contributes to dysbiosis, compromising the integrity of the colon barrier and increasing the presence of toxic metabolites and proteolytic activity. These factors collectively contribute to inflammation and the onset of CRC.^45^ Specifically, Uchino et al. identified significantly higher levels of *P. stomatis*, *S. anginosus*, *S. moorei*, and *S. koreensis* in both saliva and the digestive tract of CRC patients.^38^ These findings align with the observations of Flemer et al., who noted elevated levels of periodontal pathogens like *F. nucleatum* and *Porphyromonas gingivalis* in the tumor tissues of CRC patients.^12^ Additionally, Pignatelli et al. reported the presence of *F. nucleatum* in both the oral cavity and matched colon cancer tissue.^58^ These studies collectively support the notion that these oral bacteria can migrate to the gastrointestinal tract, triggering inflammation, altering the host immune response, and creating an environment conducive to tumor growth.^45^

The potential of oral microbiota to migrate via the circulatory system (hematogenous route) or through the ingestion of food and fluids into the digestive system (enteral route) suggests their presence in the gastrointestinal tract and potential involvement in carcinogenesis.^59^ Animal models have proposed three potential mechanisms for bacterial migration: (1) disruption of gut homeostasis leading to intestinal bacterial overgrowth, (2) increased permeability of the intestinal mucosal barrier, and (3) deficiencies in host immune defenses. However, the exact transmission route remains uncertain, and conclusive evidence on the subject is currently lacking.^60^

The majority of the studies took into consideration the stage of CRC (Table 2). Uchino et al. categorized CRC into early (stage I, II) and advanced stages (stage III, IV), revealing that *Solobacterium moorei* was present at a significantly higher relative abundance in the advanced‐stage group compared to the early‐stage group.^38^ This suggests that *S. moorei* could be associated not only with CRC carcinogenesis but also CRC progression by creating an inflammatory environment.^61^ In a similar context, *Peptostreptococcus stomatis*, *S. koreensis*, *Solobacterium moorei*, and *Streptococcus anginosus* exhibited significantly higher relative abundance in the advanced stage of the disease.^38^

Another relevant issue to consider is the sampling method used in the different studies. The various sampling sites create specific niches for microbial colonization characterized by different oxygen levels, nutrient availability, and mechanical stress conditions.^62^ Saliva represents the preferred sampling site to obtain oral microbiota DNA for processing since it tends to reflect the microbiota from all oral sites and the associated diseases.^63^ In the present systematic review, the majority of authors obtained samples from stimulated or unstimulated saliva. However, samples also originated from swabs (cheeks), tongue coating, and oral rinse. Jo et al. showed no differences in microbiota diversity in samples of unstimulated saliva, stimulated saliva, and oral rinse collected in specimen tubes.^64^ Finally, subgingival plaque can also be used. Theoretically, it reflects the local microbiota composition much more specifically than a salivary sample. Nevertheless, this sample collection method is more invasive and requires qualified trained staff to harvest subgingival plaque.^65^ None of the included studies utilized samples from subgingival plaque. Thus, methodological issues should be considered in the interpretation and generalization of the results.^66^

The status of periodontal health and oral hygiene is another factor to consider. Wang et al. reported a positive correlation between the abundance of salivary *Rothia* and *Streptococcus* and the oral hygiene index.^39^ Conversely, *Alloprevotella*, *Fusobacterium*, *Peptostreptoccus*, and *Prevotella* genus levels were negatively correlated with oral health index.^39^ Poor oral hygiene and periodontal health were observed more frequently in CRC patients compared to controls in three studies.^36^, ^38^, ^40^ Improving oral hygiene practices, as suggested by previous research,^25^, ^67^ may potentially reduce the abundance of mentioned bacterial species, thereby lowering the risk of these bacteria entering the intestines and possibly preventing colorectal cancer. However, only half of the studies considered this variable in their analysis, and assessment methods varied. Therefore, clinical trials employing standardized protocols and accounting for confounding variables are necessary to deepen our understanding of the association between oral microbiota and CRC.

The diversity of the oral microbiota varies geographically among populations and across different regions.^68^, ^69^ A study comparing salivary microbiomes in Alaskans, Germans, and Africans found greater similarities between native Alaskans and Germans than either group with Africans. *Firmicutes* were most abundant in Alaskans and Germans, while *Proteobacteria* dominated among Africans.^70^ Since most of the included studies were conducted in Asia, the generalization of the findings from this systematic review is limited. Hence, findings regarding ethnic or population‐specific disparities in human microbiome composition cast doubt on the applicability of microbiome‐based therapeutic interventions and underscore the need for locally customized community‐level strategies for microbiome modulation.^69^

The studies encountered a significant limitation due to the absence of standardized procedures in both sample collection and processing. Various methods, such as 16S rRNA V1‐V2, V3‐V4 and qPCR, were utilized by researchers to identify bacteria. However, significant heterogeneity existed within these methods, particularly in the selection of primers, which could potentially introduce biases and render comparisons nearly impracticable.

The concept of utilizing oral microbiota as diagnostic biomarkers for CRC holds significant promise. However, several aspects need to be considered in evaluating the feasibility, reliability, and potential influence of CRC on oral microbiota.^66^ Therefore, several challenges need to be addressed before oral microbiota‐based biomarkers can be implemented in clinical practice. In general, the evidence presented in this review is of low‐to‐moderate quality as assessed by the GRADE framework. Large‐scale prospective studies are needed to validate the diagnostic accuracy and clinical utility of oral microbiota biomarkers for CRC.

## 5 CONCLUSIONS

In conclusion, while the concept of utilizing oral microbiota as diagnostic biomarkers for CRC shows promise, it requires further research and validation to establish its feasibility, reliability, and clinical utility. Addressing key challenges and advancing our understanding of the complex interactions between oral microbiota and CRC will be essential for realizing the full potential of this approach in early detection and management of the disease.

### 5.1 Implications for clinical practice

- Certain oral bacteria, primarily *Streptococcus*, *Prevotella intermedia*, *Fusobacterium nucleatum*, and *Neisseria oralis*, along with secondarily *Lactobacillus* and *Rothia*, may be implicated in colorectal cancer evolution as potentially characteristic genera.
- Conversely, specific oral bacteria, including *Lachnospiraceae*, *Fusobacterium periodonticum*, and *Prevotella melaninogenica*, could play a protective effect against colorectal cancer.

### 5.2 Implications for research

- The detection of certain oral microbes in saliva may potentially contribute to non‐invasive prediction of colorectal cancer.
- Mechanisms affecting colorectal cancer or evolution could include triggering or worsening systemic inflammation, migration of the oral microbiota or their products via the blood vessel or via the digestive tract, and inflammatory process and molecules induced by oral dysbiosis.

## AUTHOR CONTRIBUTIONS

All authors contributed to the study's conception and design. Preliminary search was performed by Sara Camañes‐Gonzalvo. Piloting of the study selection process was performed by Carlos Bellot‐Arcís and Vanessa Paredes‐Gallardo. Formal screening of search results against eligibility criteria was performed by Sara Camañes‐Gonzalvo, Miriam Lobo‐de‐Mena, and María José Safont‐Aguilera. Data extraction and data analysis were performed by José María Montiel‐Company. Risk of bias assessment was performed by Sara Camañes Gonzalvo and Amaya Fernández‐Diaz. The first draft of the manuscript was written by Sara Camañes Gonzalvo and Andrés López‐Roldán and all authors commented on previous versions of the manuscript. All authors read and approved the final manuscript.

## FUNDING INFORMATION

This research did not receive any specific grant from funding agencies in the public, commercial, or not‐for‐profit sectors.

## CONFLICT OF INTEREST STATEMENT

The authors have no relevant financial or non‐financial interests to disclose.

## PROTOCOL AND REGISTRATION

The present systematic review was previously registered in PROSPERO under registration number CRD42023459036.

## Supporting information


Data S1.

## Data Availability

The data that support the findings of this study are available from the corresponding author upon reasonable request.
