# Supplementary material for: Relationship between oral microbiota and colorectal cancer: A systematic review
Source: J Periodontal Res. 2024 May 22;59(6):1071–82. doi: 10.1111/jre.13289 (PMC11626693; doi:10.1111/jre.13289)
Supplement: Supplementary file 1 — Data S1. [file JRE-59-1071-s001.docx]

**Relationship between oral microbiota and colorectal cancer. A systematic review.**

*Journal of Periodontal Research*

Sara Camañes-Gonzalvo; José María Montiel-Company^*^; Miriam Lobo-de-Mena; María José Safont-Aguilera; Amaya Fernández-Diaz; Andrés López-Roldán; Vanessa Paredes-Gallardo; Carlos Bellot-Arcís.

*Corresponding author:

José María Montiel-Company.

Faculty of Medicine and Dentistry. University of Valencia. C/Gascó Oliag 1 – 46010 Valencia, Spain.

Email: [jose.maria.montiel@uv.es](mailto:jose.maria.montiel@uv.es)

**Supplementary Information**

**Table S1** Electronic search strategy for the different databases.

| **PubMed (MEDLINE)** | |
| --- | --- |
| Item | Search strategy |
| #1  #2  #3  Total | (patient) OR (cancer patient)  (oral microbiota) OR (oral bacteria) OR (oral microorganisms) OR (periodontal pathogens) OR (oral pathogens) OR (periodontitis) OR (periodontal disease) OR (oral biomarker)  (colorectal cancer) OR (colonic cancer) OR (digestive cancer) OR (gastrointestinal cancer) OR (digestive tract cancer)  #1 AND #2 AND #3 |
| **Web of science** | |
| Item | Search strategy |
| #1  #2  #3  Total | ALL= “patient*” OR "cancer patient*"  ALL= "oral microbiota" OR "oral bacteria" OR "oral microorganisms" OR "periodontal pathogens" OR "oral pathogens" OR “periodontitis” OR “periodontal disease” OR “oral biomarker”  ALL= "colorectal cancer" OR “colonic cancer” OR “digestive cancer” OR “gastrointestinal cancer” OR “digestive tract cancer”  #1 AND #2 AND #3 |
| **EMBASE** | |
| Item | Search strategy |
| #1  #2  #3  ­­­­­  Total | patient*:ab,ti OR 'cancer patient*':ab,ti  'oral microbiota':ab,ti OR 'oral bacteria':ab,ti OR 'oral microorganisms':ab,ti OR 'periodontal pathogens':ab,ti OR 'oral pathogens':ab,ti OR 'periodontitis':ab,ti OR 'periodontal disease':ab,ti OR ' oral biomarker ':ab,ti  'colorectal cancer':ab,ti OR 'colonic cancer':ab,ti OR 'digestive cancer':ab,ti OR 'gastrointestinal cancer':ab,ti OR 'digestive tract cancer':ab,ti  #1 AND #2 AND #3 |
| **SCOPUS** | |
| Item | Search strategy |
| #1  #2  #3  Total | TITLE-ABS-KEY ("patient" OR "cancer patient")  TITLE-ABS-KEY ("oral microbiota" OR "oral bacteria" OR "oral microorganisms" OR "periodontal pathogens" OR "oral pathogens" OR “periodontitis” OR “periodontal disease” OR “oral biomarker”)  TITLE-ABS-KEY ("colorectal cancer" OR “colonic cancer” OR “digestive cancer” OR “gastrointestinal cancer” OR “digestive tract cancer”)  #1 AND #2 AND #3 |

**Table S2** Quality of observational studies assessed using the Newcastle-Ottawa scale for case-control studies.

| **ARTICLE** | **SELECTION**  **1 2 3 4** | **COMPARABILITY**  **5a 5b** | **EXPOSURE**  **6 7 8** | **TOTAL** |
| --- | --- | --- | --- | --- |
| Kato et al., 2016 [29] | * * * * |  | * * * | 7/9 |
| Han et al., 2016 [30] | * * * * |  | * * * | 7/9 |
| Russo et al., 2017 [31] | * * * * | * * | * * | 8/9 |
| Yamamura et al., 2017 [32] | * * | * | * * * | 6/9 |
| Flemer et al., 2018 [12] | * * * * | * | * * * | 8/9 |
| Guven et al., 2019 [33] | * * * * | * * | * * * | 9/9 |
| Kageyama et al., 2019 [34] | * * * * | * * | * * * | 9/9 |
| Schmidt et al., 2019 [35] | * * * * | * | * * * | 8/9 |
| Yang et al., 2019 [36] | * * * * | * | * * * | 8/9 |
| Zhang et al., 2020 [37] | * * * * | * | * * * | 8/9 |
| Uchino et al., 2021[38] | * * * * | * | * * * | 8/9 |
| Wang et al., 2021[39] | * * * * | * * | * * * | 9/9 |
| Idrissi Janati et al., 2022 [40] | * * * * | * | * * * | 8/9 |
| Morsi et al., 2022 [41] | * * * * | * | * * * | 8/9 |
| Zhang et al., 2022 [42] | * * * * | * | * * * | 8/9 |
| Rezasoltani et al., 2023 [22] | * * | * * | * * * | 7/9 |

***Criteria****: Selection (1. Is the case definition adequate? 2. Representativeness of cases, 3. Selection of cases, 4. Definition of controls). Comparability (5. comparability of cases and controls on the basis of the design analysis for the most important confounder 5a. more important and 5b. for other factors). Exposure (6. Exposure assessment, 7. Same verification method for cases and controls, 8. Nonresponse rate).*

**Figure S1** Quality of observational studies assessed using the Newcastle-Ottawa scale for case-control studies expressed as a graphic.


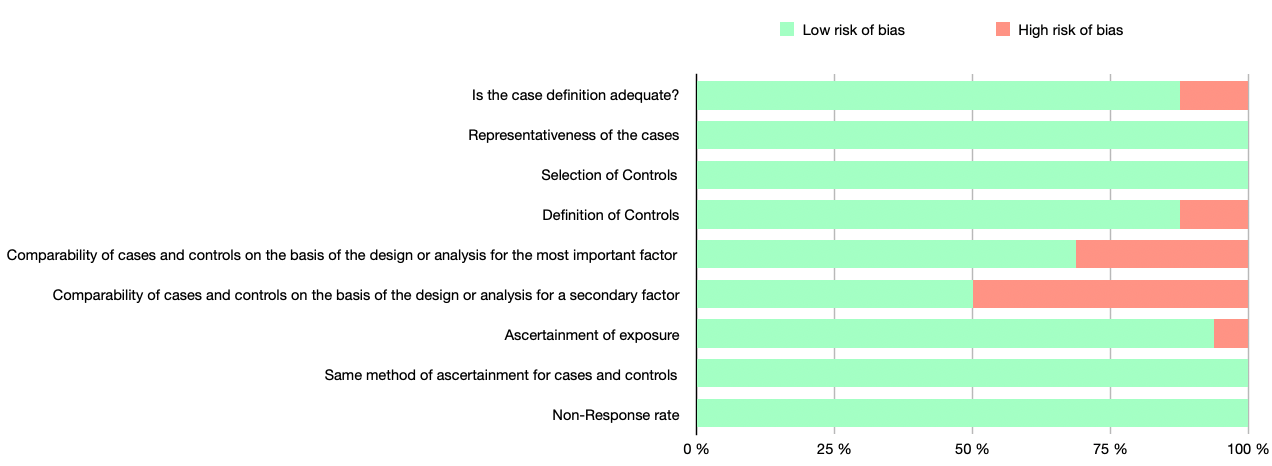


**Table S3** Grading of Recommendation, Assessment, Development, and Evaluation (GRADE) analysis.

| **Certainty of assessment** | | | | | | | **Certainty** |
| --- | --- | --- | --- | --- | --- | --- | --- |
| Nº of studies | Study design | Risk of bias | Inconsistency | Indirectness | Imprecision | Other considerations |  |
| **Increased levels of *Streptococcus sp.* could be implicated in colorectal cancer evolution and progression** | | | | | | | |
| **6** [12, 33, 35; 38; 39; 42] | CC | Not serious | Serious* | Not serious | Not serious | None | Low-moderate  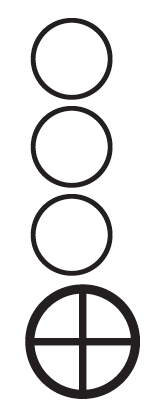 |
| **Increased levels of *Prevotella intermedia sp.* could be implicated in colorectal cancer evolution and progression** | | | | | | | |
| **5** [12, 30, 36, 37, 39] | CC | Not serious | Serious* | Not serious | Not serious | None | Low 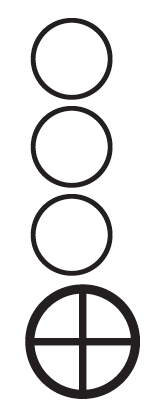 |
| **Increased levels of *Fusobacterium nucleatum sp.* could be implicated in colorectal cancer evolution and progression** | | | | | | | |
| **7** [12, 22, 31; 33; 38; 40; 41] | CC | Not serious | Serious* | Not serious | Not serious | None | Low-moderate  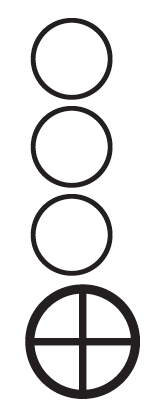 |
| **Increased levels of *Neisseria oralis sp.* could be implicated in colorectal cancer evolution and progression** | | | | | | | |
| **4** [12, 34; 36; 39] | CC | Not serious | Serious* | Not serious | Not serious | None | Low  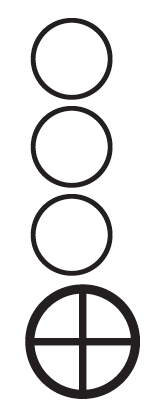 |
| **Increased levels of *Lactobacillus* *sp.* could be implicated in colorectal cancer evolution and progression** | | | | | | | |
| **2** [29, 36] | CC | Not serious | Serious* | Not serious | Not serious | None | Very Low  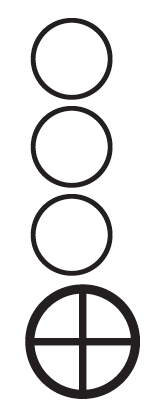 |
| **Increased levels of *Rothia* *sp*. could be implicated in colorectal cancer evolution and progression** | | | | | | | |
| **2** [12, 29] | CC | Not serious | Serious* | Not serious | Not serious | None | Very Low  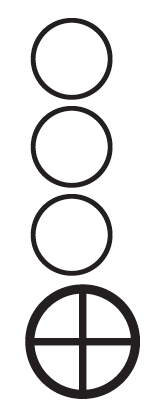 |
| ***Lachnospiraceae sp.* could play a protective role against colorectal cancer** | | | | | | | |
| **1** [12] | CC | Not serious | Not serious | Not serious | Not serious | None | Very Low  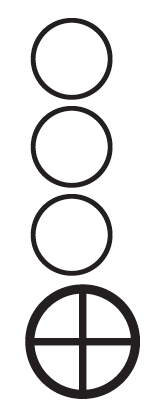 |
| ***Fusobacterium periodonticum sp.* could play a protective role against colorectal cancer** | | | | | | | |
| **2** [30, 39] | CC | Not serious | Not serious | Not serious | Not serious | None | Very Low  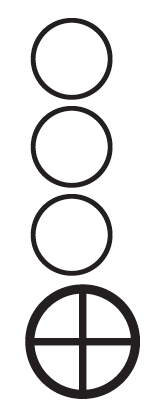 |
| ***Prevotella melaninogenica.* could play a protective role against colorectal cancer** | | | | | | | |
| **2** [34, 36] | CC | Not serious | Not serious | Not serious | Not serious | None | Very Low  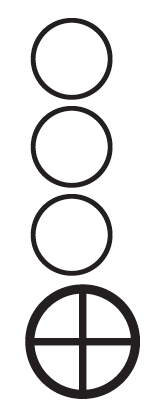 |

*There is some heterogeneity in the study sample, follow-up, and assessment method.
